# Effect of Environmental Exposures on the Gut Microbiota from Early Infancy to Two Years of Age

**DOI:** 10.3390/microorganisms9102140

**Published:** 2021-10-13

**Authors:** Kameron Y. Sugino, Tengfei Ma, Nigel Paneth, Sarah S. Comstock

**Affiliations:** 1 Department of Food Science and Human Nutrition, Michigan State University, East Lansing, MI 48824, USA; kameron-sugino@ouhsc.edu; 2 Department of Epidemiology and Biostatistics, College of Human Medicine, Michigan State University, East Lansing, MI 48824, USA; matengfe@msu.edu (T.M.); paneth@msu.edu (N.P.); 3 Department of Pediatrics and Human Development, College of Human Medicine, Michigan State University, East Lansing, MI 48824, USA

**Keywords:** infant, toddler, gut microbiota, microbiome, human milk, obesity, longitudinal cohort

## Abstract

The gut microbiota undergoes rapid changes during infancy in response to early-life exposures. We have investigated how the infant gut bacterial community matures over time and how exposures such as human milk and antibiotic treatment alter gut microbiota development. We used the LonGP program to create predictive models to determine the contribution of exposures on infant gut bacterial abundances from one month to two years of age. These models indicate that infant antibiotic use, human milk intake, maternal pre-pregnancy BMI, and sample shipping time were associated with changes in gut microbiome composition. In most infants, *Bacteroides*, *Lachnospiraceae* unclassified, *Faecalibacterium*, *Akkermansia*, and *Phascolarctobacterium* abundance increased rapidly after 6 months, while *Escherichia*, *Bifidobacterium*, *Veillonella*, and *Streptococcus* decreased in abundance over time. Individual, time-varying, random effects explained most of the variation in the LonGP models. Multivariate association with linear models (MaAsLin) displayed partial agreement with LonGP in the predicted trajectories over time and in relation to significant factors such as human milk intake. Multiple factors influence the dynamic changes in bacterial composition of the infant gut. Within-individual differences dominate the temporal variations in the infant gut microbiome, suggesting individual temporal variability is an important feature to consider in studies with a longitudinal sampling design.

## 1. Introduction

During the first 2–3 years of life, the gut microbiome undergoes rapid and important changes to bacterial community structure and function [1,2]. This period of maturation is characterized by early abundances of *Bifidobacterium, Bacteroides*, and *Escherichia*, which are gradually replaced by obligate anaerobic bacteria, notably members of the Firmicutes phylum, such *Clostridiaceae* and *Lachnospiraceae* [3]. One of the strongest drivers of infant gut composition is human milk in the infant diet [1]. Human milk exposure is associated with increased *Bifidobacterium* abundance and decreased abundance of the Firmicute *Lachnospiraceae* [4,5]. Formula-fed infants tend to be enriched in *Bacteroides, Escherichia, Enterobacteriaceae, Clostridium* [1,6], and other bacteria associated with a more mature microbiota [7]. Some evidence suggests bacterial colonization in the gut by breastmilk bacteria acts in a dose-dependent manner. Pannaraj et al. found that infants whose diet consisted of >75% human milk receive around 27% of their gut bacteria from their mother’s milk, while infants who breastfeed less than that receive only about 17% of their bacteria from milk—this difference in bacterial acquisition decreases as the infants age and are exposed to other sources of bacteria [8].

Infant gut colonization is also influenced by other exposures, including C-section delivery [9], antibiotic use [10], and maternal body mass index (BMI) [11]. Like formula feeding, these factors have been associated with early gut maturation (i.e., higher abundance of Firmicutes), which may have adverse effects on immune system development [12,13] and may increase the risk of obesity in children [11,14].

Since alteration in the gut microbiota may be linked to childhood health and development, understanding these bacterial differences and how they change over time during early development opens the door to ameliorating developmental trajectories through manipulation of bacterial communities. Here, we use longitudinal fecal microbiota data collected from children from about 1 month to 2 years of age to describe gut community changes over time and to examine how common infant exposures affect changes in taxa abundance. We found that within-individual differences dominate temporal variations in the infant gut microbiome, and that human milk exposure is a key factor. Excluding the significant results for age, only *Lachnospiraceae* unclassified and low abundances of *Bilophila*, *Butyricimonas*, and *Pyramidobacter* were associated with more than one exposure variable of interest. When comparing the LonGP results to the MaAsLin linear models, there was partial agreement between the results.

## 2. Materials and Methods

### 2.1. Study Participants

The participants for this study were enrolled as part of the ARCH_GUT_ or BABY_GUT_ cohorts. ARCH_GUT_ participants were enrolled as a sub-study of the Archive for Research in Child Health (ARCH) study. Both ARCH_GUT_ and BABY_GUT_ have been described previously [15]. Briefly, participants provided written informed consent to obtain an enrollment questionnaire (pre-pregnancy height and weight, antibiotic use in the past year, parity, diagnosed or suspected food allergies/intolerances) and fecal samples from the women during their third trimester of pregnancy and fecal samples from their infant at 1 month, 6 months, 12 months, and 24 months of age. All subjects gave their informed consent for inclusion before they participated in the study. The study was conducted in accordance with the Declaration of Helsinki, and the Michigan State University Human Research Protection Program approved these studies (IRB C07-1201, 15-1240, and 14-170M).

### 2.2. Sample Collection, DNA Extraction, and Amplification

Fecal samples were collected from infants at 1 month, 6 months, 12 months, and 24 months of age. The protocols have been described previously [16]. Briefly, samples were sent to our lab by mail or retrieved from the participant’s home. Samples were collected from diapers and placed into Para Pak collection tubes by the parent (Meridian Biosciences, Cincinnati, OH, USA). Fecal aliquots were stored at −80 °C upon reaching the lab. DNA extractions were performed using the MoBio Powersoil DNA Isolation kit (Qiagen MoBio, Carlsbad, CA, USA) and the V4 region of the 16S rRNA gene was amplified using primer sets SB501-SB508 and SA701-SA712 following the mothur wet lab SOP [17]. Successful amplification triplicates were pooled and purified using Agencourt AMPure XP (Beckman Coulter, Brea, CA, USA). Equal amounts (in nanograms) of the purified 16S samples were pooled and submitted to the Michigan State University Research Technology Support Facility Genomics Core for paired-end 250 base-pair sequencing on the Illumina MiSeq platform using V2 chemistry.

### 2.3. Processing and Analysis of Sequence Data

Sequence reads were processed in mothur using the Illumina MiSeq SOP [17]. Operational taxonomic unit (OTU) taxonomies were assigned by phylotype in mothur using the SILVA reference taxonomy (release V132) [18]. Read processing was done in mothur using the High-Performance Computing Cluster at Michigan State University. Sample reads were rarefied to 10,000 reads for 999 times, averaged, and rounded to the nearest integer before further analysis. Rarefaction curves were generated to confirm adequate community coverage.

### 2.4. Statistical Analysis

We applied the “Envfit” function in the “vegan” package to identify the variables significantly associated with the gut microbial community at each collection period. The “Envfit” function fits the environmental vectors onto an ordination, which is a 2D plot that displays how similar/dissimilar one sample’s bacterial community is compared to other samples. Ordination for “Envfit” was also performed in the “vegan” package using non-metric multidimensional scaling (NMDS) methods with Bray–Curtis dissimilarity. We included the following 9 variables that have been found related to gut microbiome development in neonates: breastfeeding status, sample shipping time, maternal pre-pregnancy BMI, antibiotic exposure at the time of sampling, antibiotic exposure since birth, sex, delivery mode, and cohort. In our analysis, we did not differentiate between samples that were shipped versus not shipped to the lab. However, these data were captured by the “shipping time” variable, where samples that were not shipped have a “shipping time” value of 0 or 1 days (*n* = 9), while the samples that were shipped to the lab have a value of 2 days or greater (*n* = 154). *p*-values calculated from “Envfit” were adjusted by the false discovery rate (FDR) method (Benjamini–Hochberg procedure [19]) for multiple comparison.

To determine the contribution of each independent variable to the changes of the infant gut microbiota, we used LonGP [20], an additive Gaussian process regression. LonGP implements time-varying random effects and non-stationary signals by integrating multiple kernel learning. We included all the variables in the “Envfit” analysis as fixed effects and child age as an individual-specific time-varying random effect. LonGP then selects variables that significantly improve the models’ prediction for each bacterial abundance. Variable information and kernel type for each variable used in LonGP are specified in Table S1. We modeled the top 50 most abundant genera separately with the same independent variables. The OTU counts were log transformed for the analysis, and the scale was back transformed for interpretation of the data. Continuous covariates were centered to the mean to generate appropriate priors for each function. 

To validate the results of LonGP, we applied multivariate association with linear models (MaAsLin) [21] to investigate the association between exposures and individual OTUs. We performed this multivariate statistical framework in the R package “MaAsLin2” [22] with the default parameters. Differences in participant characteristics between the two cohorts used in this study were tested using the chi-squared test, ANOVA, or Spearman correlation where appropriate. Only significant associations with a *q*-value ≤ 0.05 after FDR correction (Benjamini–Hochberg procedure) were included.

## 3. Results

The population characteristics for all infant samples are shown in Table 1. There were no significant differences in participant characteristics between the two cohorts. Characteristics for the participants with a sample at every time point (*n* = 34) are shown in Table S2. We first examined the association between the single variables and gut microbial community beta diversity at each collection period using the “Envfit” function in the vegan package (Table 2). Only maternal pre-pregnancy BMI and breastfeeding status were significantly associated with gut microbiota at 6 months of age.

**Figure 1:**
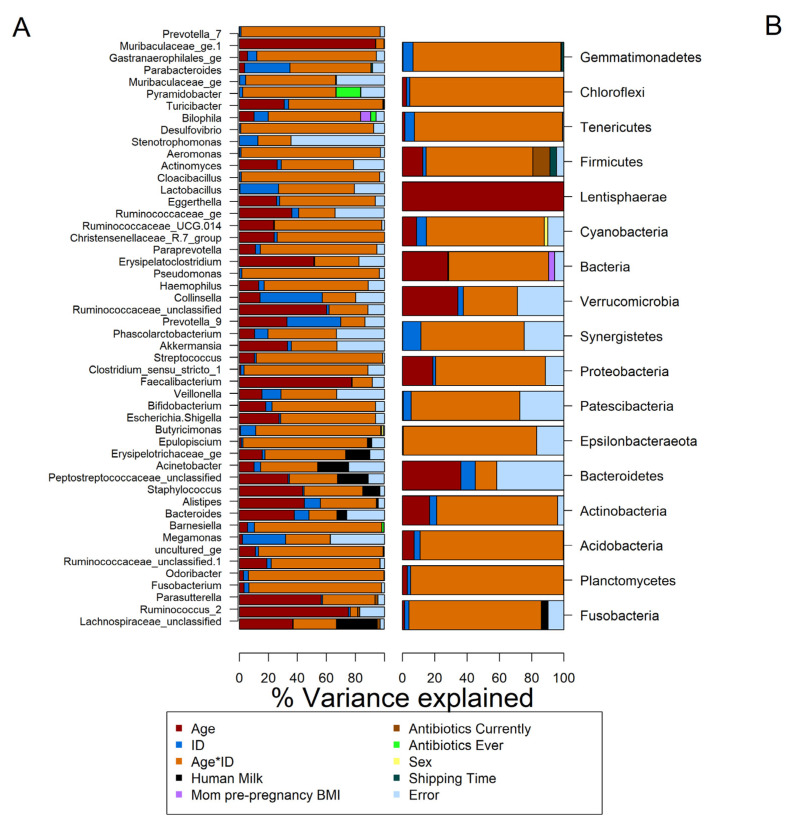
Model parameters significantly associated with the predicted abundance at the genus (**A**) and phylum (**B**) levels. The figure also illustrates the variance in the predicted abundance of a single genera or phyla explained by each parameter.

We then performed the longitudinal analysis (using LonGP) for the gut microbiota data collected at 1 month, 6 months, 12 months, and 24 months. Figure 1 shows the model parameters that are predictive of each genera’s abundance. The individual time-varying random effect (age*ID) explained most of the variation identified in this longitudinal dataset. The extent of human milk feeding was significant in 12 genera, infant antibiotic exposure at the time of sampling was significant for 7 genera, sample shipping time in 6 models, “antibiotic exposure ever” was significant in 4 models, maternal pre-pregnancy BMI was significant in 2 models, and sex in 1 model (Figure 1A). At the phylum level, 9 phyla were significantly associated with at least one variable in addition to age and id. Within these 9 phyla, 5 were associated with shipping, 3 were associated with antibiotic exposure at the time of sampling, 2 were associated with human milk feeding, 2 with maternal BMI, and 1 with sex (Figure 1B).

**Figure 2:**
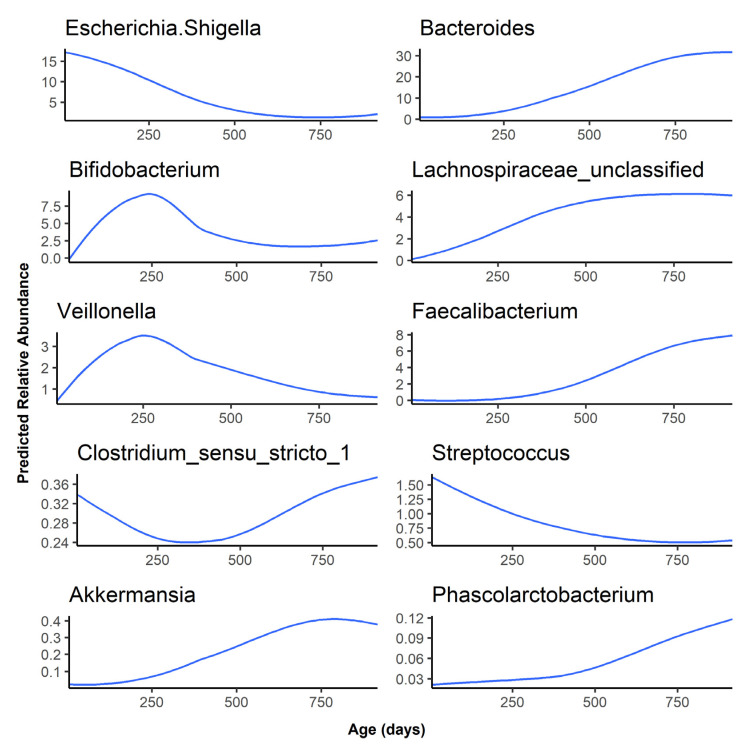
Predicted abundance over time for the top 10 most abundant genera.

As the infants aged, *Bacteroides*, *Lachnospiraceae* unclassified, *Faecalibacterium*, *Akkermansia*, and *Phascolarctobacterium* abundance increased rapidly after 6 months, while *Escherichia*, *Bifidobacterium*, *Veillonella*, and *Streptococcus* decreased in abundance as the infant gut microbiota matured over time (Figure 2). Finally, *Clostridium sensu stricto* 1 was predicted to have a relative abundance of less than 0.5% at all timepoints and followed a bimodal distribution, with a higher abundance at the 1-month timepoint, a sharp decrease in abundance at 6 and 12 months, followed by an abundance comparable to the 1-month timepoint at 24 months. The age association for the remaining 40 genera examined can be found in Figure S1. Of these, 31 started at a low abundance and increased as the infant aged. However, genera such as *Staphylococcus* and *Lactobacillus* started at a higher abundance before decreasing over time.

The predicted relative abundance of each taxon is shown as a function of infant age. Note that the scale of the y-axis differs for each bacterium.

**Figure 3:**
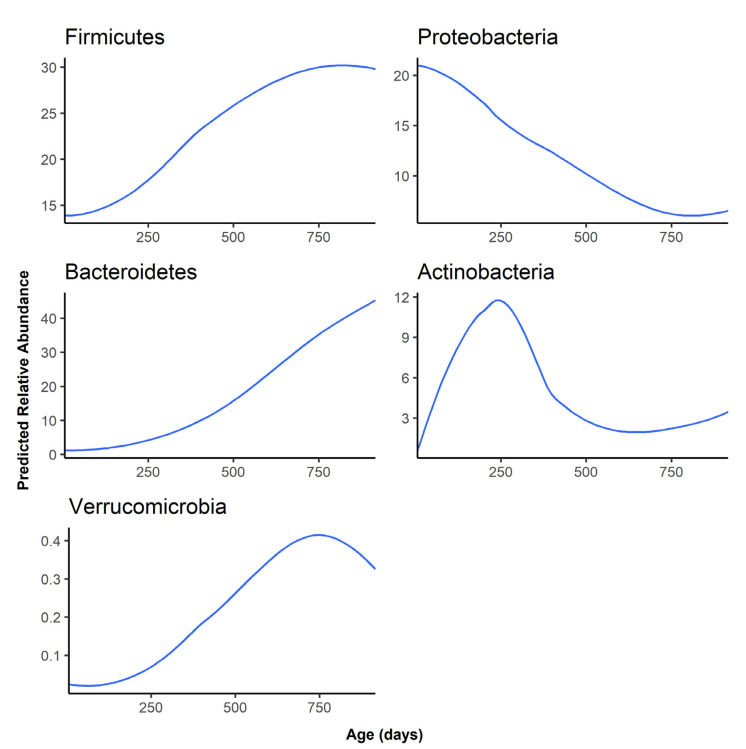
Predicted abundance over time for the top 5 most abundant phyla.

At the phylum level, Firmicutes and Bacteroidetes became the most abundant phyla as the infant aged, replacing Proteobacteria as the dominant gut phylum by one year of age (Figure 3). Besides Firmicutes, Bacteroidetes, and Proteobacteria, the remaining phyla were all predicted to have an abundance of lower than 1% at all time points (Figure S2).

The predicted relative abundance of each taxon is shown as a function of infant age. Note that the scale of the y-axis differs for each phylum.

Infants who were taking antibiotics at the time of fecal sampling were predicted to have lower abundances of bacteria such as *Lachnospiraceae* unclassified, *Ruminococcaceae*, and the phylum Firmicutes (Figures S4 and S5). The genus *Fusobacterium* and phylum Lentisphaerae were both predicted to have a lower abundance in infants taking antibiotics at the time of fecal sampling. Infants who had ever taken antibiotics had higher predicted abundances of *Bilophila*, *Megamonas*, *Barnesiella*, and *Pyrimidobacter* (Figure S6).

**Figure 4:**
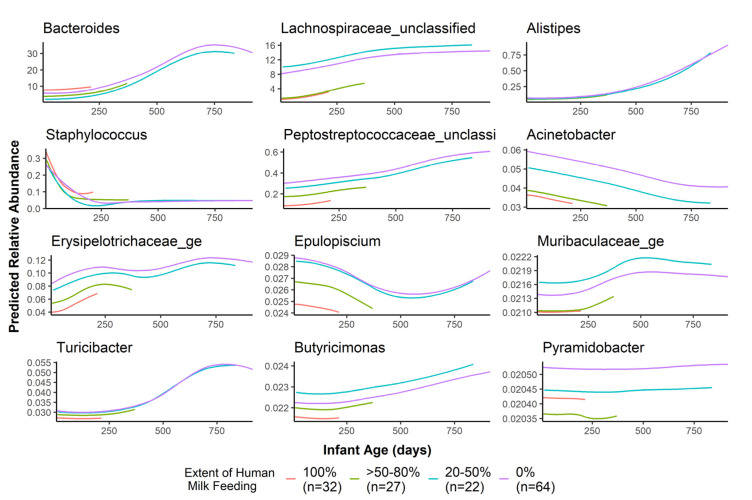
Genera relative abundance as predicted by age and human milk in the infant diet. Only those genera significantly predicted by age and human milk intake are plotted.

Human milk exposure was predictive of 12 genera (Figure 4) and 2 phyla (Figure S3). Notably, infants who consumed <50% human milk had higher abundances of several bacteria such as *Lachnospiraceae* unclassified, *Peptostreptococcaceae* unclassified, and *Acinetobacter*. Unlike the other taxa, *Bacteroides* was predicted to be highest in the exclusive human milk and no human milk group, and lower in the mixed-feeding groups.

Maternal BMI was associated with two low-abundance genera (*Bilophila* and *Turicibacter*) and two low-abundance phyla (unidentified Bacteria and Gemmatimonadetes) (Figures S7 and S8) in the gut microbiota of the children.

Sample shipping time was associated with 6 genera. More time spent between sampling and arrival in the lab was predictive of increases in *Parabacteroides*, *Gastranaerophilales_ge*, *Prevotella*_7, and other low-abundance genera. Two unidentified families of Muribaculaceae decreased as the shipping time increased (Figure S9). At the phylum level, four of the five taxa associated with shipping time were predicted to have a low abundance at all time points. However, the Firmicutes phylum was predicted to increase slightly in abundance before decreasing in relative abundance by around 7% after 3–4 days of shipping before stabilizing (Figure S10).

Sex was predictive of *Butyricimonas* and Cyanobacteria, where girls were predicted to have higher abundances of both taxa compared to boys (Figures S11 and S12).

### Comparison of LonGP Genus-Level Results to MaAsLin Linear Models

As demonstrated by the MaAsLin results, infant age was significantly associated with 19 different bacterial genera (Table S3). *Ruminococcus* 2, *Faecalibacterium*, *Turicibacter*, *Alistipes*, and *Peptostreptococcaceae* unclassified are the five most significant genera that increased with infant age. *Escherichia* and *Acinetobacter* are the only two genera that decreased with infant age. Comparing the linear trend of MaAsLin to the predicted trajectories of LonGP, *Parasutterella* is the only genus that exhibited a different predicted trend. Using LonGP, *Parasutterella* was predicted to increase in abundance from 6 months to 24 months and then decrease (Figure S3), whereas MaAsLin predicted that the abundance of *Parasutterella* increases over time.

When considering variables other than age and participant, there was partial agreement between LonGP and MaAsLin only for the human milk exposure. Both models detected a significant relationship between human milk exposure and abundance of *Bacteroides*, *Staphylococcus*, *Lachnospiraceae* unclassified, *Peptostreptococcaceae* unclassified, *Erysipelotrichaceae*, *Acinetobacter*, and *Epulopiscium*. Many of the associations agreed with each other between the two models. For example, for the taxa that overlap between the models, both found that >50% human milk feeding had the lowest predicted abundances of these taxa except for *Staphylococcus*, which was highest in the exclusively human milk-fed group in both models. For *Bacteroides*, both models found that 80% human milk feeding had the lowest predicted abundance of this genus compared to exclusive human milk feeding.

## 4. Discussion

Our longitudinal analysis of the infant gut microbiota aimed to identify key exposures that play a role in shaping the infant microbiome from one month to two years of age. Using a program that is specifically designed for statistical analysis of longitudinal data, LonGP, we found that age is the strongest predictor of microbiota change over the course of infancy, followed by the extent of human milk in the diet, a finding consistent with other studies [23]. Excluding the significant results for age, only *Lachnospiraceae* unclassified and the sparse *Bilophila*, *Butyricimonas*, and *Pyramidobacter* were associated with more than one exposure variable of interest. When comparing the LonGP results to the MaAsLin linear models, we observed agreement between the results. However, the MaAsLin models tended to identify more bacteria as significantly associated with the exposure variables than LonGP identified.

A previous study of the gut microbiota in adults demonstrated that between-individual differences can explain up to 61% variation in community composition and that between-individual difference is much more pronounced than within-individual difference because the highly diverse gut microbiota of adults is very stable over time [24]. However, our longitudinal sampling of infants has shown that the time-varying random effect (within-individual temporal variability) dominates the changes in the infant gut microbiome and explain up to 95% of the variation in individual taxa. The higher variation explained by within-individual differences in infants can be explained by the rapid maturation of the infant gut microbiota over the first year of life as the community transitions to an adult-like profile by 3 years of age [25]. Our results confirmed that the dynamics of human microbiome composition are a personalized feature [26] and are important to consider in a longitudinal analysis of the gut microbiome.

In this cohort, we observed a dose–response relationship between the extent of milk in the infant diet and abundances of several taxa, including *Bacteroides* and *Lachnospiraceae*. *Bacteroides* is a genus of common gut microbes whose members are able to metabolize HMOs in breastmilk [6] as well as assist in the transition of the microbiome from formula or human milk to solid foods [27]. Our results show that the highest predicted levels of *Bacteroides* are in the exclusively human milk-fed group and the group with no human milk in the diet, displaying the metabolic flexibility of this genus and its importance as an early colonizer of the infant gut [28]. In our cohort, *Lachnospiraceae* unclassified was associated with human milk intake and antibiotic exposure at the time of fecal sampling. *Lachnospiraceae* abundance generally increases with infant age [5,29], and is a common taxon associated with gut microbiome maturation [30]. Similar to other studies [5], we found that infants receiving <50% human milk in their diet had significantly higher levels of *Lachnospiraceae* unclassified. *Lachnospiraceae* abundance in 3-month-old infants has been associated with increased risk for childhood overweight at 1 year of age [14], though the mechanisms behind this association are not known. However, it is hypothesized that short-chain fatty acid production (mainly acetate, propionate, and butyrate) by members of the *Lachnospiraceae* family contribute to obesity risk by upregulating lipogenesis [31] and altering immune system responses to the normal gut flora and their metabolites [32,33]. Our results suggest that an infant diet low in human milk influences the abundance of important members of the infant gut, which have previously been associated with increased infant risk for obesity development.

For this study, we categorized antibiotic exposure as antibiotic use during the time of fecal sample collection or any use of antibiotics before fecal sample collection. As others have reported [34], we found that antibiotic use at the time of sampling depleted some bacterial abundances. However, if the participant had ever been exposed to antibiotics, they were predicted to have higher abundances of *Megamonas*, *Bilophila*, *Barnesiella,* and *Pyramidobacter*. This shift may represent opportunistic growth of certain bacteria after perturbation with antibiotics. For example, *Bilophila* is a potential opportunistic pathogen that has been investigated for its role in promoting inflammation [35] and has been implicated in the development of insulin resistance and induction of metabolic dysfunction under high-fat-diet conditions [36]. *Bilophila* also displayed a unique pattern of association with maternal pre-pregnancy BMI. Our models predicted a small increase in abundance at a maternal BMI of 25 followed by a large increase in predicted abundance as maternal BMI increased past 30. This suggests that infants born to women with class 2 or 3 obesity (corresponding to a BMI of 35+) may be at a higher risk of developing metabolic syndrome and insulin resistance in a diet-dependent manner. High abundance of *Bilophila* may also predispose these infants to higher levels of intestinal inflammation by making the epithelium more permeable to other microbial compounds such as lipopolysaccharides [37], which can cause further systemic inflammation [38].

Interestingly, neither LonGP nor MaAsLin identified delivery mode as a significant factor at either the genus or phylum levels. Most studies report that differences in the microbiome attributable to mode of delivery disappear by around 6 months of age [1,39,40], though a more recent study found differences in bacterial abundances up to 1 year of age [41]. Since microbiome differences due to delivery mode are tied to age and gut maturation, studies that explore microbiome differences by delivery mode may not be able to utilize methods such as LonGP or MaAsLin for longitudinal analysis of the data, but instead would need to analyze each timepoint separately to observe the effects of delivery mode on bacterial community composition.

Several taxa abundances were predicted to change as the sample shipping time was extended. The most abundant taxon that changed with shipping time was the phylum Firmicutes, which is composed of obligate anaerobic bacteria, and was predicted to increase slightly in abundance before quickly depleting after 3–4 days. Interestingly, none of the genera predicted to change with shipping time are members of the phylum Firmicutes. Since no genera within the Firmicutes phylum were specifically associated with shipping time, but the Firmicutes phylum was, this suggests that Firmicutes depletion was evenly distributed across all genera in the phylum—leading to little absolute change per genus—or the variability of the Firmicutes across timepoints was greater than the variability due to shipping, preventing the detection of significant shifts in specific genera. Shipping samples at room temperature is known to affect microbiota abundances [42,43,44], but community differences across samples are preserved regardless of storage method [43,45,46]. In other words, ensuring that samples are all processed using the same method is potentially more important than immediate sample processing since differing storage conditions result in unique biases in the data.

There are several strengths and limitations to this study. First, the sample collection method was not ideal, since immediate freezing is the gold-standard followed by refrigeration or storage in a preservative during transit [42]. This could have affected the bacterial abundances in our samples and may explain some of our results, such as a lack of significance between *Bifidobacterium* abundance and human milk exposure. We also did not consider timing of complementary feeding in our analysis. Introduction of complementary foods is associated with increased gut maturity and a more diverse functional profile [47], meaning early introduction of complementary foods may have had short- and long-term impacts on gut microbiota development that we were not able to differentiate from age or extent of breastfeeding. Strengths include sample collection during a critical developmental window and a more granular look at the impact of different levels of human milk exposure.

## 5. Conclusions

In summary, we characterized the infant gut microbiota over the first two years after birth and found age and extent of human milk feeding were the strongest predictors of microbiome change over this period. Notably, low levels of human milk feeding were associated with increased abundances of *Lachnospiraceae*, which may increase infant risk of childhood overweight/obesity later in life [14]. Similarly, antibiotic exposure and maternal obesity were associated with an increase in abundance of *Bilophila*, an opportunistic pathogen that may affect insulin resistance and alter metabolic responses to a high-fat, Western-style diet [36]. We have shown that the abundances of several gut microbes are associated with infant exposures as well as maternal pre-pregnancy BMI. More research needs to be done to further describe how early alterations to certain microbial taxa—as well as their metabolic products—can shape microbiome compositions later in life and whether these changes affect the risk of developing chronic diseases, such as allergies and obesity.

## Figures and Tables

**Table 1:** Population characteristics of the infants at 1, 6, 12, and 24 months.

	1 Month			6 Months			12 Months			24 Months		
	ARCH_GUT_	BABY_GUT_	*p*-Value	ARCH_GUT_	BABY_GUT_	*p*-Value	ARCH_GUT_	BABY_GUT_	*p*-Value	ARCH_GUT_	BABY_GUT_	*p*-Value
*n*	27	16		25	16		24	16		22	17	
Vaginal Delivery ^1^	18 (66.7)	10 (62.5)	1	17 (68.0)	9 (56.2)	0.67	16 (66.7)	10 (62.5)	1	15 (68.2)	10 (58.8)	0.79
Girls ^1^	12 (44.4)	3 (18.8)	0.17	10 (40.0)	3 (18.8)	0.28	9 (37.5)	3 (18.8)	0.36	7 (31.8)	3 (17.6)	0.53
Infant breastmilk ^1^			0.28			0.47			0.12			0.35
100	15 (55.6)	13 (81.2)		3 (12.0)	4 (25.0)		0 (0)	0 (0)		0 (0)	0 (0)	
>50–80	4 (14.8)	2 (12.5)		11 (44.0)	8 (50.0)		0 (0)	2 (12.5)		0 (0)	0 (0)	
20–50	6 (22.2)	1 (6.2)		1 (4.0)	1 (6.2)		9 (37.5)	3 (18.8)		1 (4.5)	2 (11.8)	
0	2 (7.4)	0 (0)		10 (40.0)	3 (18.8)		15 (62.5)	11 (68.8)		21 (95.5)	14 (82.4)	
Missing	0 (0)	0 (0)		0 (0)	0 (0)		0 (0)	0 (0)		0 (0)	1 (5.9)	
Antibiotic exposure currently ^1^	0 (0)	2 (12.5)	0.26	0 (0)	0 (0)	1	2 (8.3)	1 (6.2)	1	0 (0)	0 (0)	1
Antibiotic exposure ever ^1^	1 (3.7)	1 (6.2)	1	1 (4.0)	2 (12.5)	0.69	8 (33.3)	4 (25.0)	0.83	9 (40.9)	4 (23.5)	0.42
Maternal pre-pregnancy BMI ^2^	29.4 ± 5.4	27.2 ± 5.9	0.24	29.6 ± 5.5	26.9 ± 6.0	0.15	29.5 ± 5.5	27.2 ± 5.9	0.21	29.8 ± 5.7	26.9 ± 5.8	0.14
Infant age (days) ^2^	38.5 ± 32.0	36.7 ± 33.7	0.86	204.9 ± 15.7	208.6 ± 32.4	0.68	385.5 ± 17.4	386.2 ± 22.0	0.9	749.6 ± 21.7	767.1 ± 49.0	0.19
Infant age (days) ^3^	26 (6, 116)	21 (9, 125)	0.83	204.0 (179, 239)	198.5 (163, 295)	0.7	383 (356, 432)	379 (364, 438)	0.55	746 (712, 800)	746 (723, 916)	0.51
Sample shipping time (days) ^2^	4.7 ± 2.8	5.5 ± 3.9	0.48	5.6 ± 4.4	3.6 ± 1.9	0.05	4.4 ± 2.6	5.2 ± 7.1	0.66	4.4 ± 3.6	4.6 ± 3.8	0.88
Sample shipping time (days) ^3^	4 (1, 14)	4 (1, 15)	0.69	5 (1, 22)	3 (0, 7)	0.14	4 (1, 11)	3.5 (2, 31)	0.55	3 (1, 18)	4 (0, 14)	0.95

^1^*n* (%); ^2^ mean ± SD; ^3^ median (min, max). BMI, body mass index.

**Table 2:** Correlation between selected variables and the gut bacterial community beta diversity.

Variable	1 Month		6 Months		12 Month		24 Month	
	R2	*p*-Value	R2	*p*-Value	R2	*p*-Value	R2	*p*-Value
Maternal pre-pregnancy BMI	0.170	0.171	0.231	0.031	0.080	0.446	0.023	0.946
Delivery mode	0.040	0.327	0.007	0.99	0.040	0.446	0.022	0.946
Child sex	0.009	0.705	0.003	0.99	0.015	0.699	0.009	0.946
Breastfeeding status	0.083	0.427	0.331	**<0.001**	0.016	0.822	0.028	0.946
Cohort	0.020	0.517	0.045	0.447	0.004	0.995	0.005	0.946
Antibiotic exposure at the time of sampling	0.047	0.327	0	1	0.281	0.338	0	1
Antibiotic exposure since birth	0.066	0.262	0.009	0.990	0.050	0.496	0.036	0.946
Sample shipping time	0.075	0.327	0.052	0.729	0.041	0.446	0.021	0.946

Significant *p* values (*p* < 0.05) are reported in bold. BMI, body mass index.

## Data Availability

The data are not publicly available due to privacy concerns given the small sample size. However, the data presented in this study are available upon request from the corresponding author.

## Supplementary Materials

The following are available online at https://www.mdpi.com/article/10.3390/microorganisms9102140/s1, Table S1: Variables implemented in the LonGP with variable explanation and kernel type, Table S2. Population characteristics of infants with all four samples, Table S3: significant MaAsLin results for the 50 most abundant taxa, Figure S1. Predicted abundance as the infant ages for the 40 least abundant genera, Figure S2 Predicted abundance as the infant ages for the 12 least abundant phyla, Figure S3. Phylum relative abundance predicted by age and human milk in the infant diet, Figure S4 Genus relative abundance predicted by age and antibiotic use at the time of fecal sampling, Figure S5 Phylum relative abundance predicted by age and antibiotic use at the time of fecal sampling, Figure S6 Genus relative abundance predicted by age and antibiotic use ever, Figure S7 Genus relative abundance predicted by maternal pre-pregnancy BMI, Figure S8 Phylum relative abundance predicted by maternal pre-pregnancy BMI, Figure S9 Genus relative abundance predicted by sample shipping time, Figure S10 Phylum relative abundance predicted by sample shipping time, Figure S11 Genus relative abundance predicted by age and sex, Figure S12 Phylum relative abundance predicted by age and sex.
