# Supplementary material for: Effect of Environmental Exposures on the Gut Microbiota from Early Infancy to Two Years of Age
Source: Microorganisms. 2021 Oct 13;9(10):2140. doi: 10.3390/microorganisms9102140 (PMC8537618; doi:10.3390/microorganisms9102140)
Supplement: Supplementary file 1 [file microorganisms-09-02140-s001.zip › microorganisms-1395634-supplementary.pdf]

Supplementary

Table S1: Variables implemented in the LonGP with variable explanation and kernel type

Variable name	Variable explanation	Kernel type
age	Child age at sampling	Squared exponential kernel
ship	Sample shipping time	Non-stationary kernel
mombmi	Maternal pre-pregnancy BMI	Squared exponential kernel
abxcurrently	Antibiotic exposure at the time of sampling	Non-stationary kernel
abxever	Antibiotic exposure since birth	Binary Kernel
bf	Breastfeeding status	Non-stationary (ns) kernel
cohort	Cohort	Binary Kernel
rout	Delivery mode	Binary Kernel
sex	Child sex	Binary Kernel
id[1]	Individual ID	Categorical kernel
id*age[2]	interaction term for Individual ID and age	Categorical kernel * Squared exponential kernel

[1] Individual ID variable represents baseline differences between different individuals in the model.

[2] This interaction term represents individual time-varying random effect.

Table S2. Population characteristics of infants with all four samples

	1 Month	6 Months	12 Months	24 Months
**n**	34	34	34	34
**Vaginal Delivery**[1]	22 (64.7)			
**Girls**	10 (29.4)			
**Infant breastmilk**				
**100**	22 (64.7)	6 (17.6)	0 (0)	0 (0)
**>50-80**	6 (17.6)	16 (47.1)	2 (5.9)	0 (0)
**20-50**	6 (17.6)	2 (5.9)	11 (32.4)	2 (5.9)
**0**	0 (0)	10 (29.4)	21 (61.8)	31 (91.2)
**Missing**	0 (0)	0 (0)	0 (0)	1 (2.9)
**Antibiotic exposure currently**[1]	1 (2.9)	0 (0)	3 (8.8)	0 (0)
**Antibiotic exposure ever**[1]	2 (5.9)	3 (8.8)	11 (32.4)	11 (32.4)
**Maternal pre-pregnancy BMI**[2]	28.9 ± 5.9			
**Infant age**[2]	39.0 ± 32.3	204.1 ± 23.1	386.0 ± 20.5	750.2 ± 23.0
**Sample shipping time**[2]	5.4 ± 3.4	4.8 ± 3.8	4.7 ± 5.2	4.6 ± 3.8

[1]n (%)

[2]mean ± SD

Table S3: significant MaAsLin results for 50 most abundant taxa

metadata	feature	value	coef	stderr	N	pval	qval
age_in_days	Acinetobacter	age_in_days	-0.31741	0.085538	162	0.000292	0.003065
age_in_days	Akkermansia	age_in_days	0.369688	0.13294	162	0.006116	0.045868
age_in_days	Alistipes	age_in_days	0.589512	0.099991	162	2.46E-08	1.55E-06
age_in_days	Bacteroides	age_in_days	0.511948	0.12336	162	5.63E-05	0.000806
age_in_days	Bilophila	age_in_days	0.266434	0.078296	162	0.000882	0.007715
age_in_days	Christensenellaceae R.7_group	age_in_days	0.251625	0.055411	162	1.14E-05	0.000238
age_in_days	Erysipelatoclostridium	age_in_days	0.41434	0.083567	162	1.88E-06	5.39E-05
age_in_days	Escherichia.Shigella	age_in_days	-0.43714	0.111014	162	0.000125	0.001514
age_in_days	Faecalibacterium	age_in_days	0.794244	0.102827	162	1.41E-12	2.22E-10
age_in_days	Lachnospiraceae unclassified	age_in_days	0.443871	0.091501	162	3.01E-06	7.91E-05
age_in_days	Parasutterella	age_in_days	0.413565	0.106869	162	0.000162	0.001886
age_in_days	Peptostreptococcaceae unclassified	age_in_days	0.391366	0.078749	162	1.79E-06	5.39E-05
age_in_days	Prevotella_9	age_in_days	0.394624	0.083593	162	5.62E-06	0.000136
age_in_days	Ruminococcaceae_ge	age_in_days	0.283425	0.065734	162	2.94E-05	0.000488
age_in_days	Ruminococcaceae UCG.014	age_in_days	0.344501	0.078299	162	2.03E-05	0.000399
age_in_days	Ruminococcaceae unclassified	age_in_days	0.329695	0.080338	162	6.64E-05	0.00091
age_in_days	Ruminococcaceae unclassified.1	age_in_days	0.173792	0.043311	162	9.45E-05	0.00119
age_in_days	Ruminococcus_2	age_in_days	0.715051	0.073904	162	1.77E-17	5.59E-15
age_in_days	Turicibacter	age_in_days	0.321417	0.051989	162	5.76E-09	4.53E-07
bf	Acinetobacter	3_20-50%	0.710755	0.206955	162	0.000786	0.007078
bf	Acinetobacter	4_0%	0.903938	0.216972	162	5.18E-05	0.000806
bf	Bacteroides	3_20-50%	-0.83825	0.296287	162	0.005382	0.042384
bf	Bifidobacterium	2_>50-80%	0.829587	0.279164	162	0.003498	0.028255
bf	Erysipelatoclostridium	3_20-50%	0.634055	0.205908	162	0.002463	0.020415
bf	Erysipelatoclostridium	4_0%	0.571422	0.207261	162	0.006548	0.046879
bf	Erysipelotrichaceae_ge	2_>50-80%	0.459713	0.167511	162	0.006856	0.047994
bf	Erysipelotrichaceae_ge	3_20-50%	0.632108	0.183485	162	0.000749	0.00694
bf	Erysipelotrichaceae_ge	4_0%	0.68023	0.186894	162	0.000375	0.003815
bf	Faecalibacterium	4_0%	0.881628	0.255029	162	0.000709	0.006764
bf	Lachnospiraceae unclassified	2_>50-80%	0.562044	0.206106	162	0.007144	0.048921
bf	Lachnospiraceae	3_20-50%	1.487852	0.225457	162	6.48E-10	6.80E-08

	unclassified						
bf	Lachnospiraceae unclassified	4_0%	1.308719	0.226939	162	4.37E-08	2.29E-06
bf	Parasutterella	4_0%	0.949131	0.266781	162	0.000502	0.004941
bf	Peptostreptococcaceae unclassified	2_>50-80%	0.663934	0.177369	162	0.000263	0.002856
bf	Peptostreptococcaceae unclassified	3_20-50%	0.806313	0.194025	162	5.52E-05	0.000806
bf	Peptostreptococcaceae unclassified	4_0%	0.912584	0.195329	162	6.68E-06	0.00015
bf	Ruminococcaceae unclassified	2_>50-80%	0.494493	0.178969	162	0.006517	0.046879
bf	Ruminococcaceae unclassified	3_20-50%	0.848386	0.196248	162	2.89E-05	0.000488
bf	Ruminococcaceae unclassified	4_0%	0.816524	0.201551	162	8.14E-05	0.001068
bf	Staphylococcus	2_>50-80%	-0.95516	0.187923	162	1.08E-06	3.78E-05
bf	Staphylococcus	3_20-50%	-1.07547	0.205566	162	5.48E-07	2.16E-05
bf	Staphylococcus	4_0%	-1.12689	0.206917	162	2.03E-07	9.12E-06
bf	Veillonella	2_>50-80%	1.081118	0.249326	162	2.82E-05	0.000488
bf	Veillonella	3_20-50%	1.043425	0.273887	162	0.000208	0.00234
bf	Veillonella	4_0%	0.948916	0.284657	162	0.001078	0.009175
sex	Alistipes	Male	0.576308	0.198871	162	0.005855	0.044982

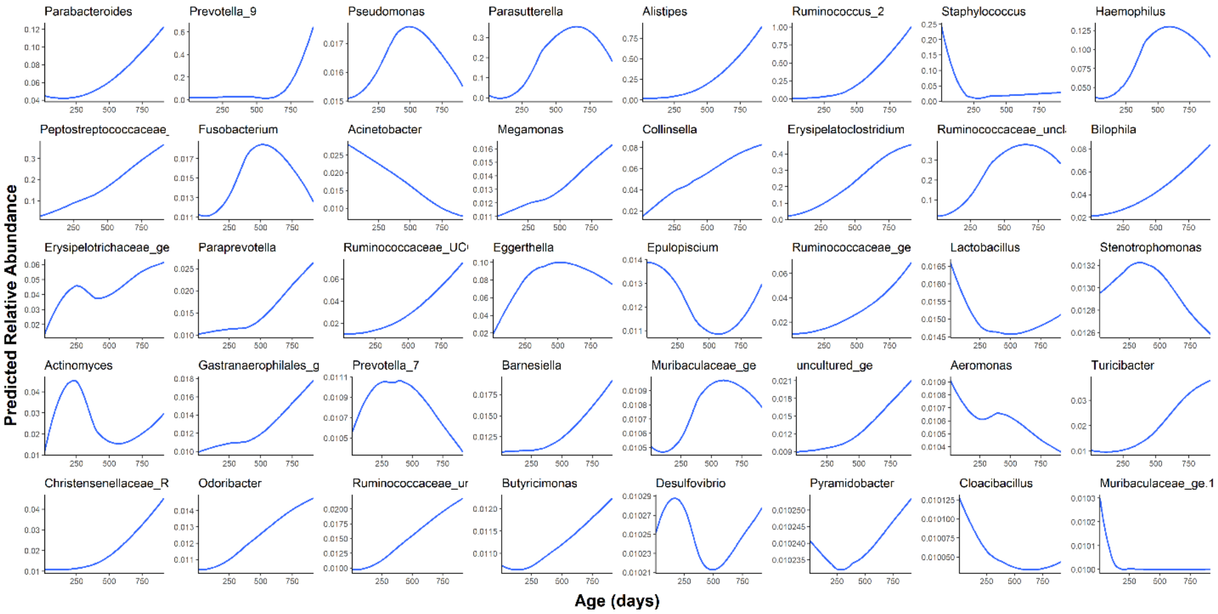

Figure S1. Predicted abundance by infant age for the 40 least abundant genera.

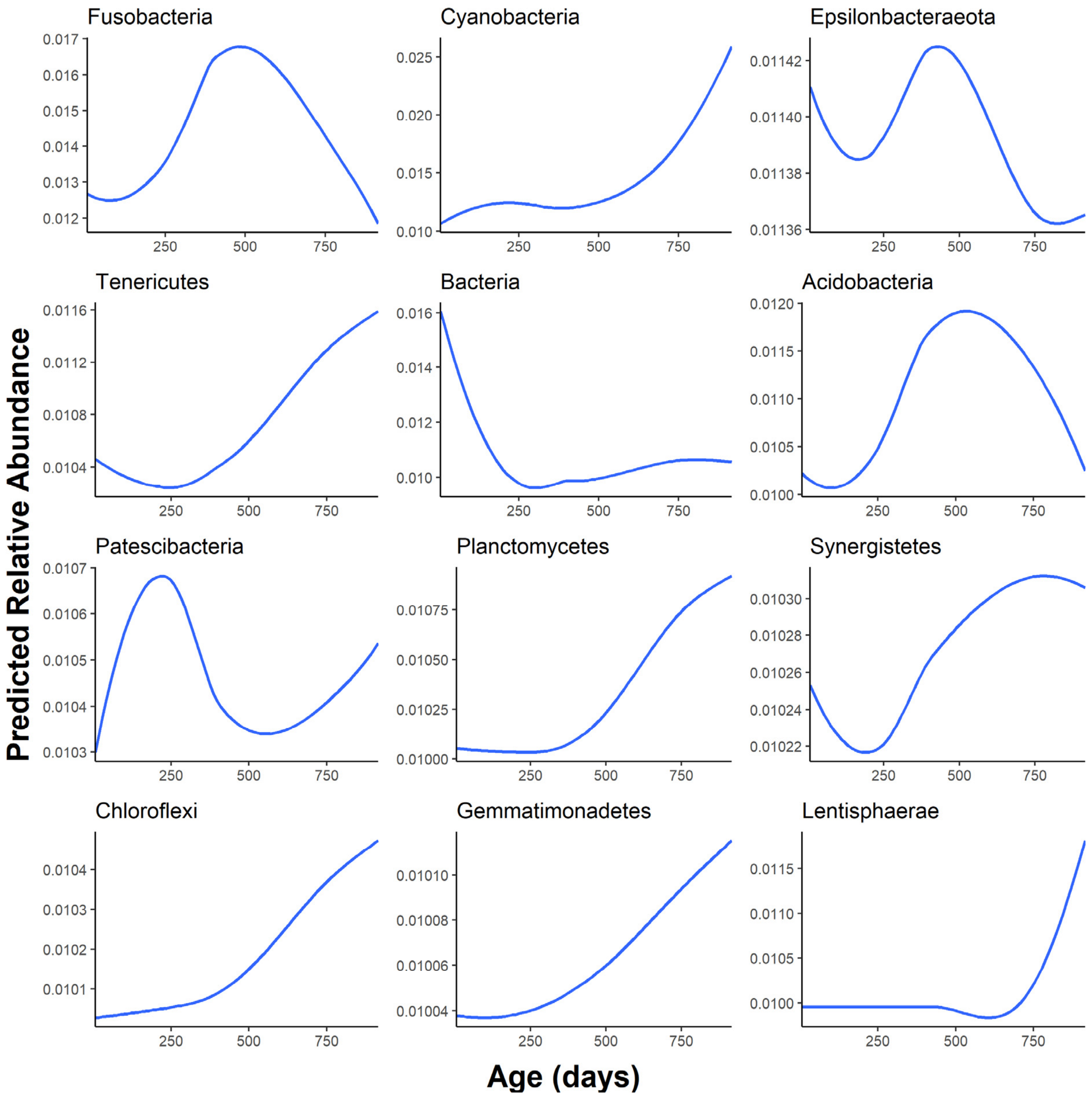

Figure S2. Predicted abundance by infant age for the 12 least abundant phyla.

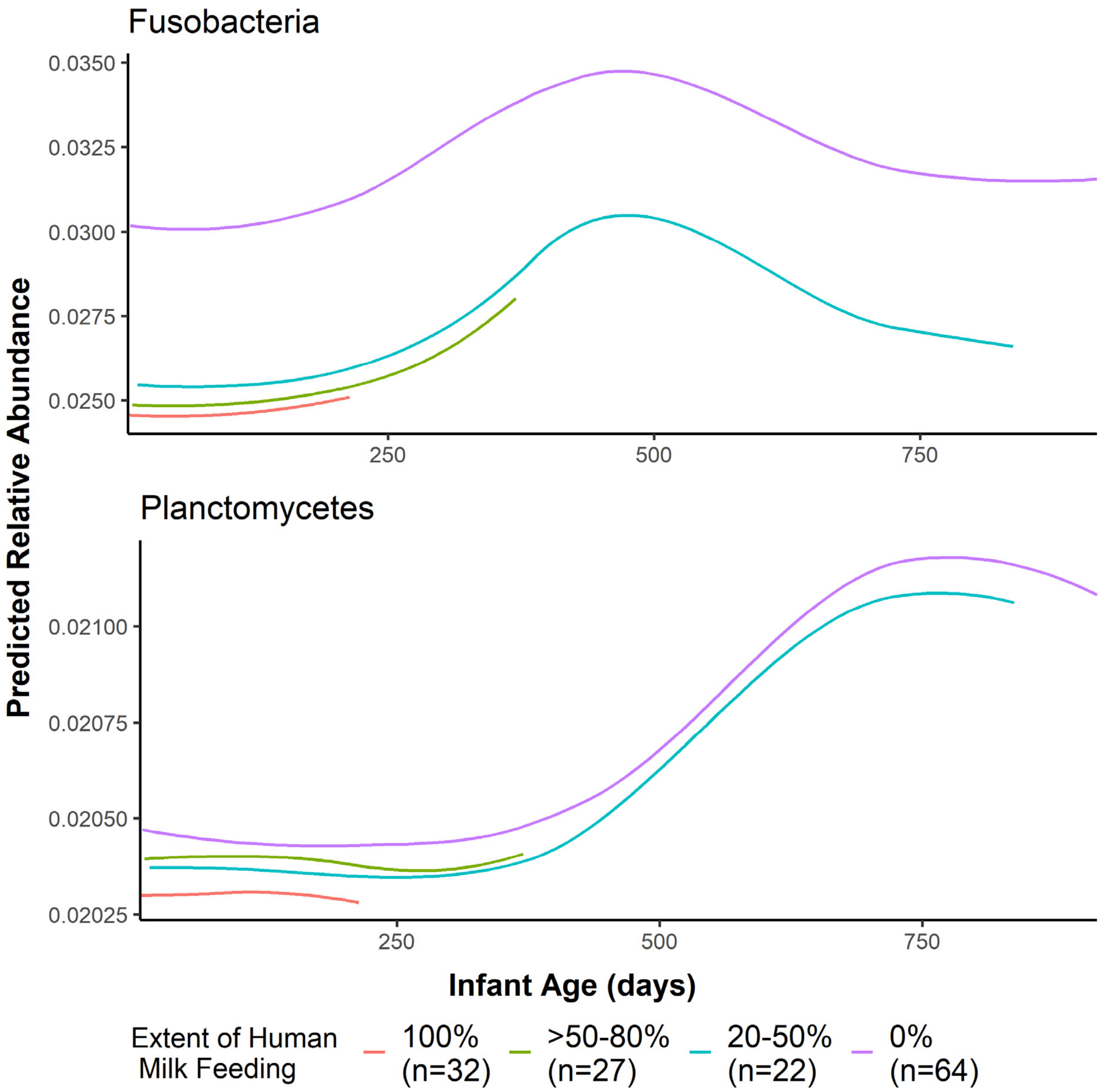

Figure S3. Phylum relative abundance predicted by age and human milk in the infant diet. Only the phyla whose abundance was significantly predicted by age and human milk are plotted.

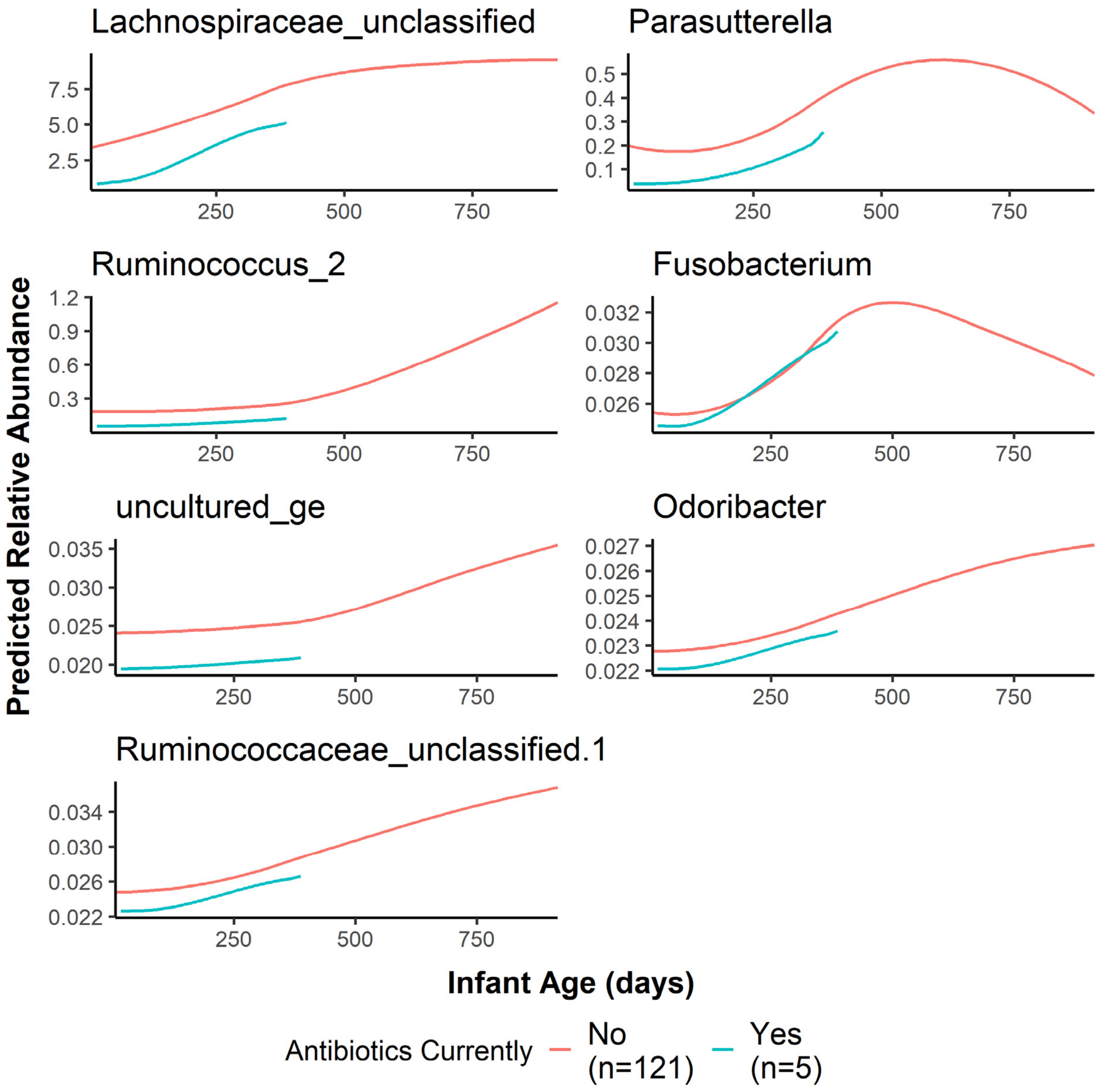

Figure S4. Genus relative abundance predicted by age and antibiotic use at the time of fecal sampling. Only the genera whose abundance was significantly predicted by age and antibiotic use at the time of sample collection are plotted.

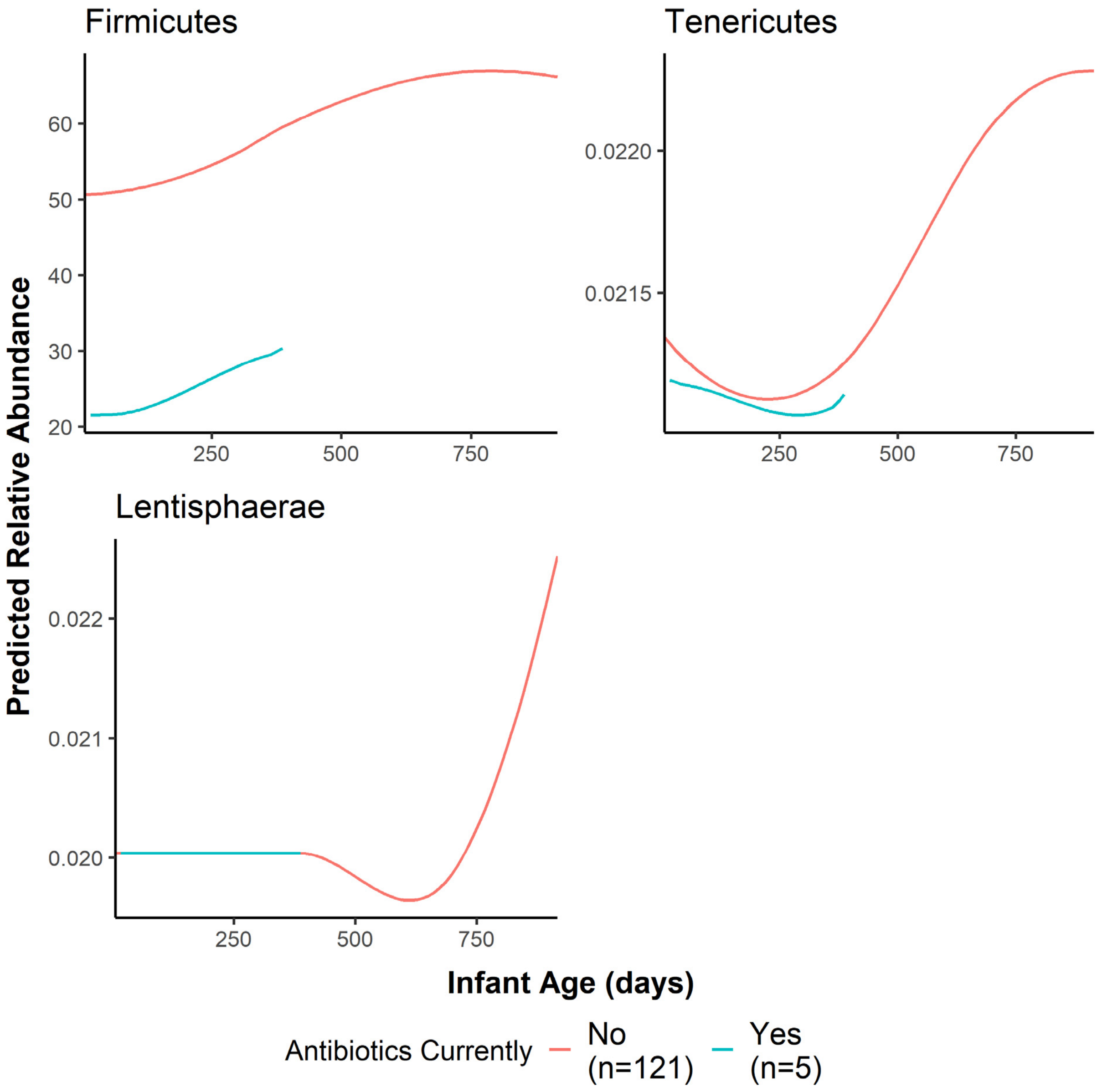

Figure S5. Phylum relative abundance predicted by age and antibiotic use at the time of fecal sampling. Only the phyla whose abundance was significantly predicted by age and antibiotic use at the time of sample collection are plotted.

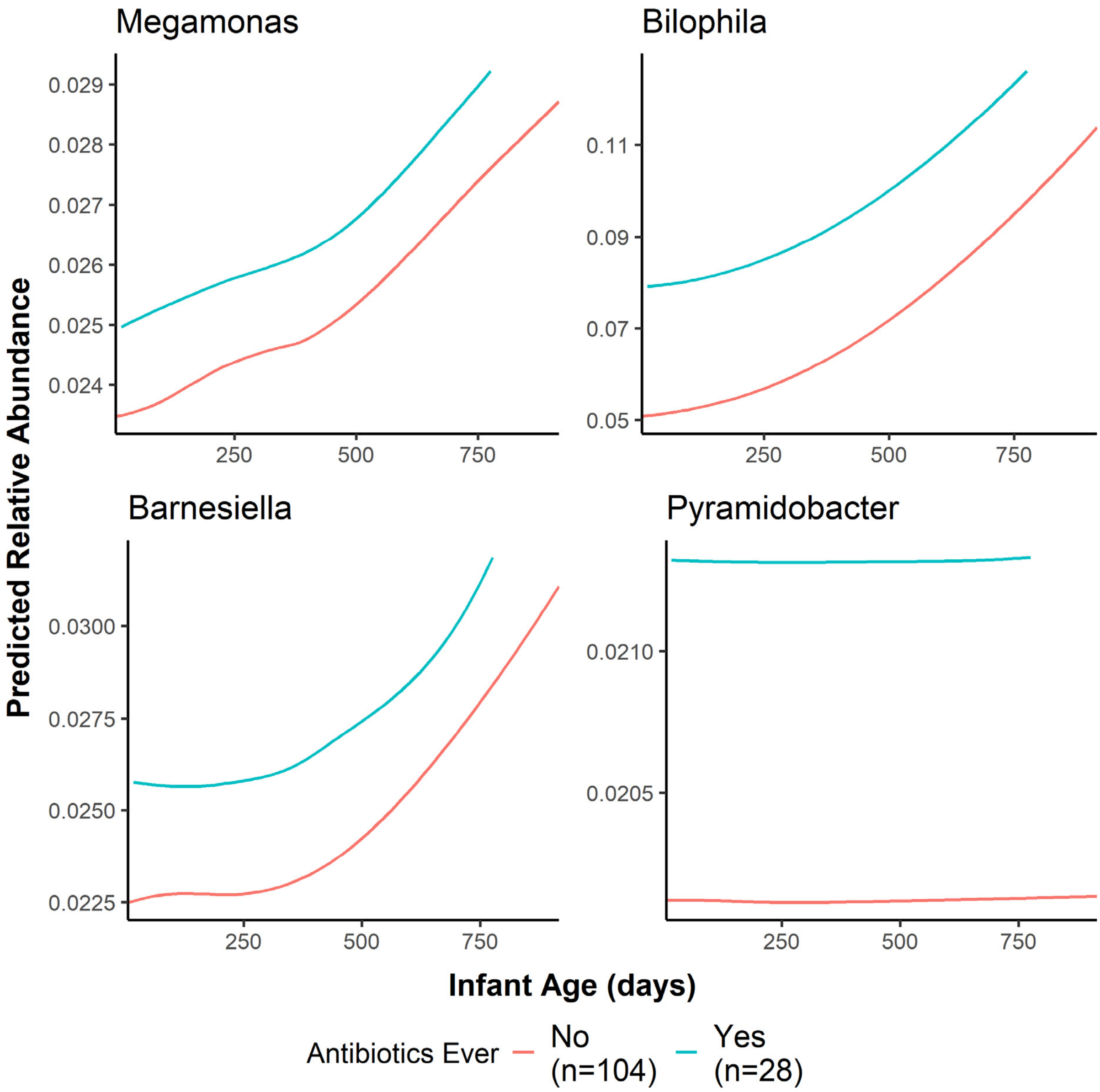

Figure S6. Genus relative abundance predicted by age and antibiotic use ever. Only the genera whose abundance was significantly predicted by age and antibiotic use ever are plotted.

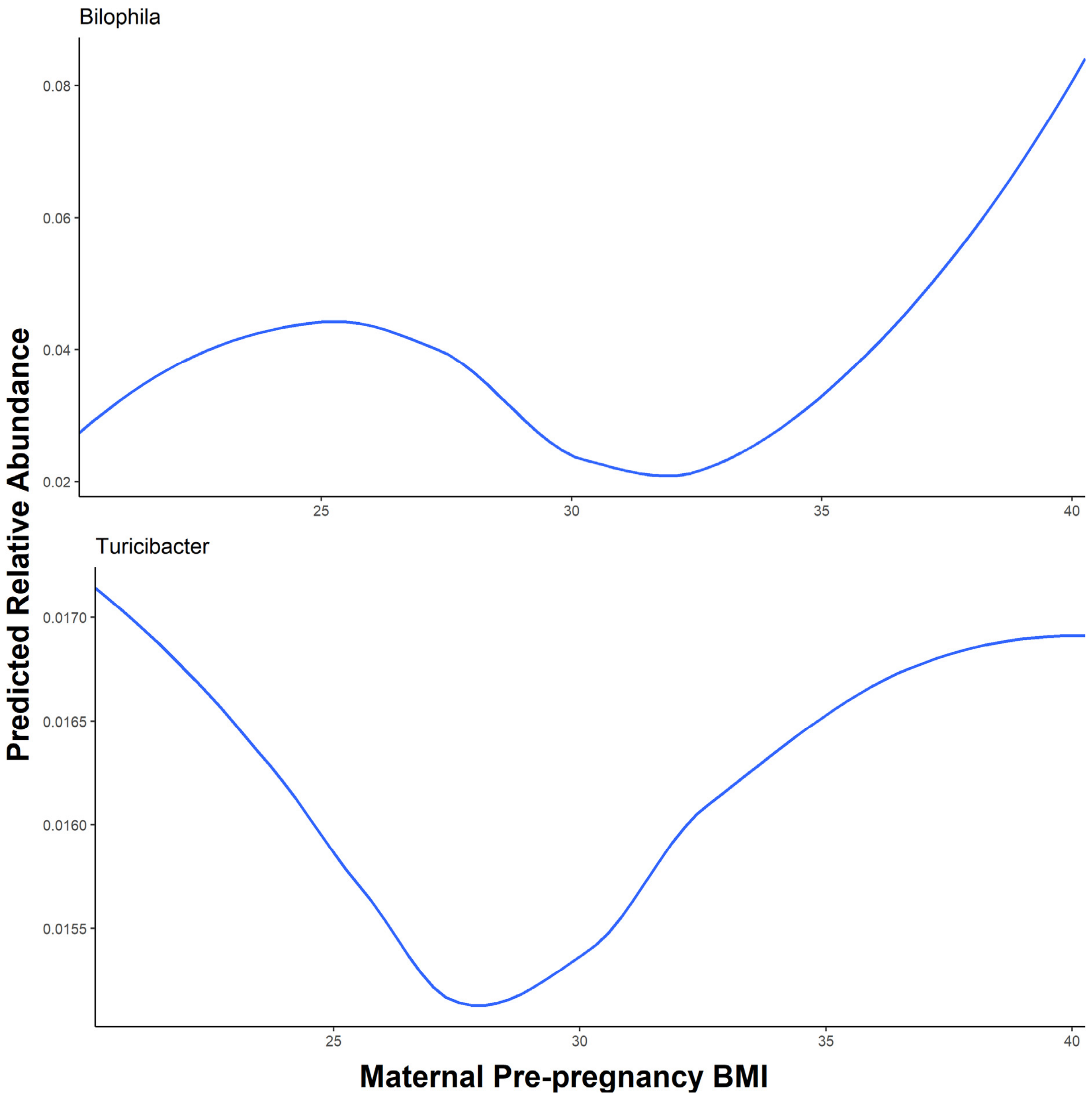

Figure S7. Genus relative abundance predicted by maternal pre-pregnancy BMI. Only the genera whose abundance was significantly predicted by age and maternal pre-pregnancy BMI are plotted.

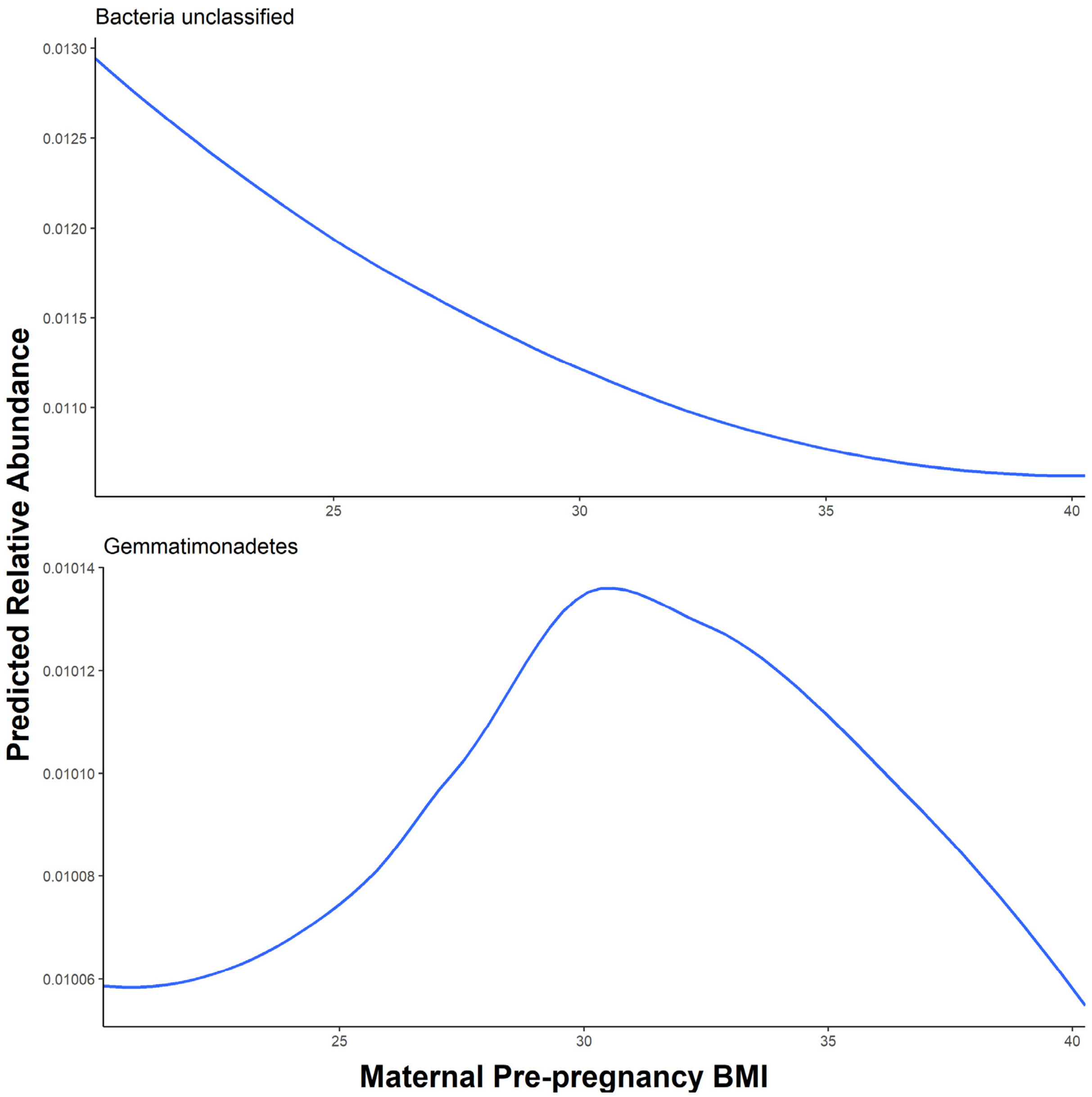

Figure S8. Phylum relative abundance predicted by maternal pre-pregnancy BMI. Only the phyla whose abundance was significantly predicted by age and maternal pre-pregnancy BMI are plotted.

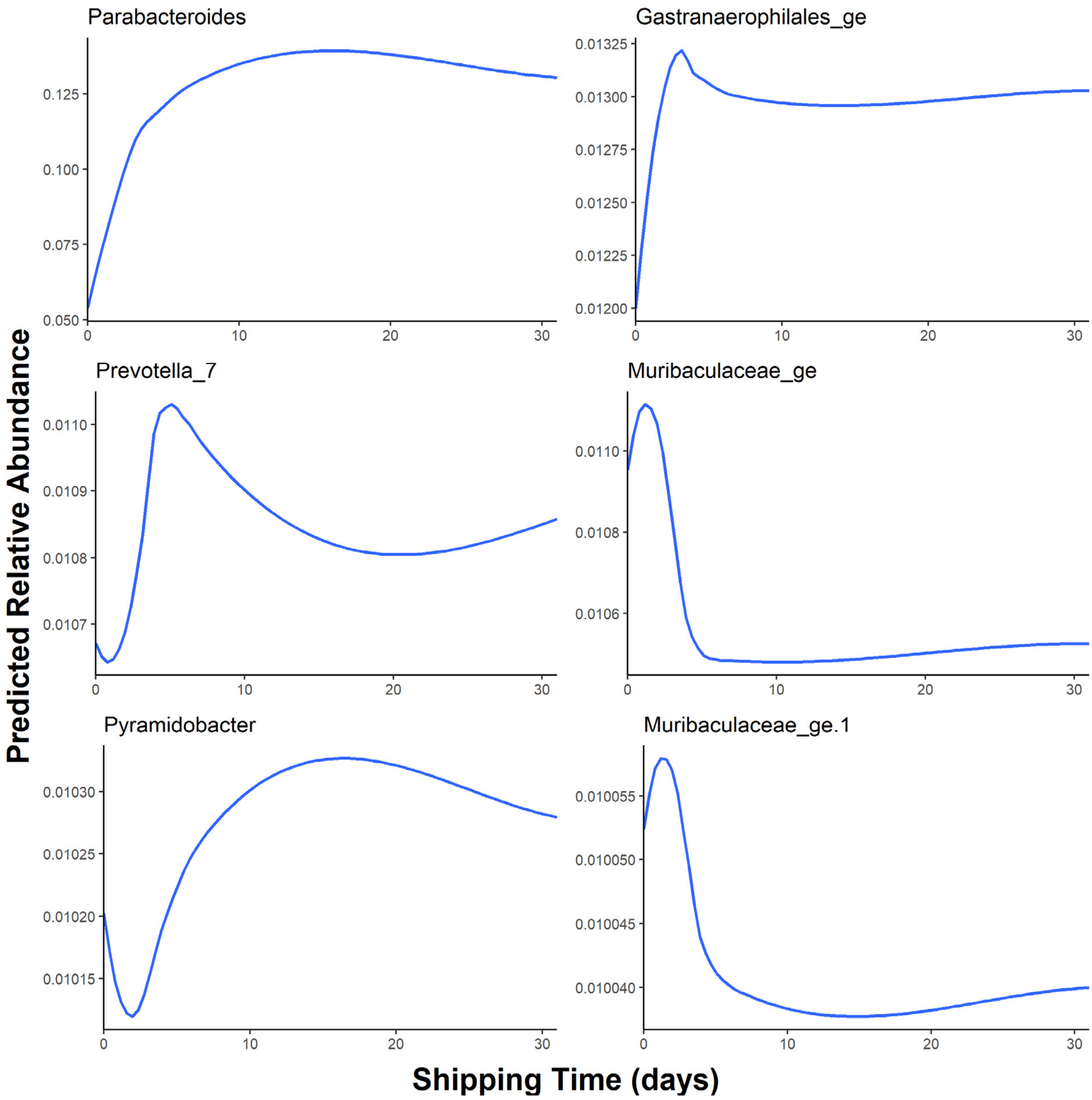

Figure S9. Genus relative abundance predicted by sample shipping time. Only the genera whose abundance was significantly predicted by age and sample shipping time are plotted.

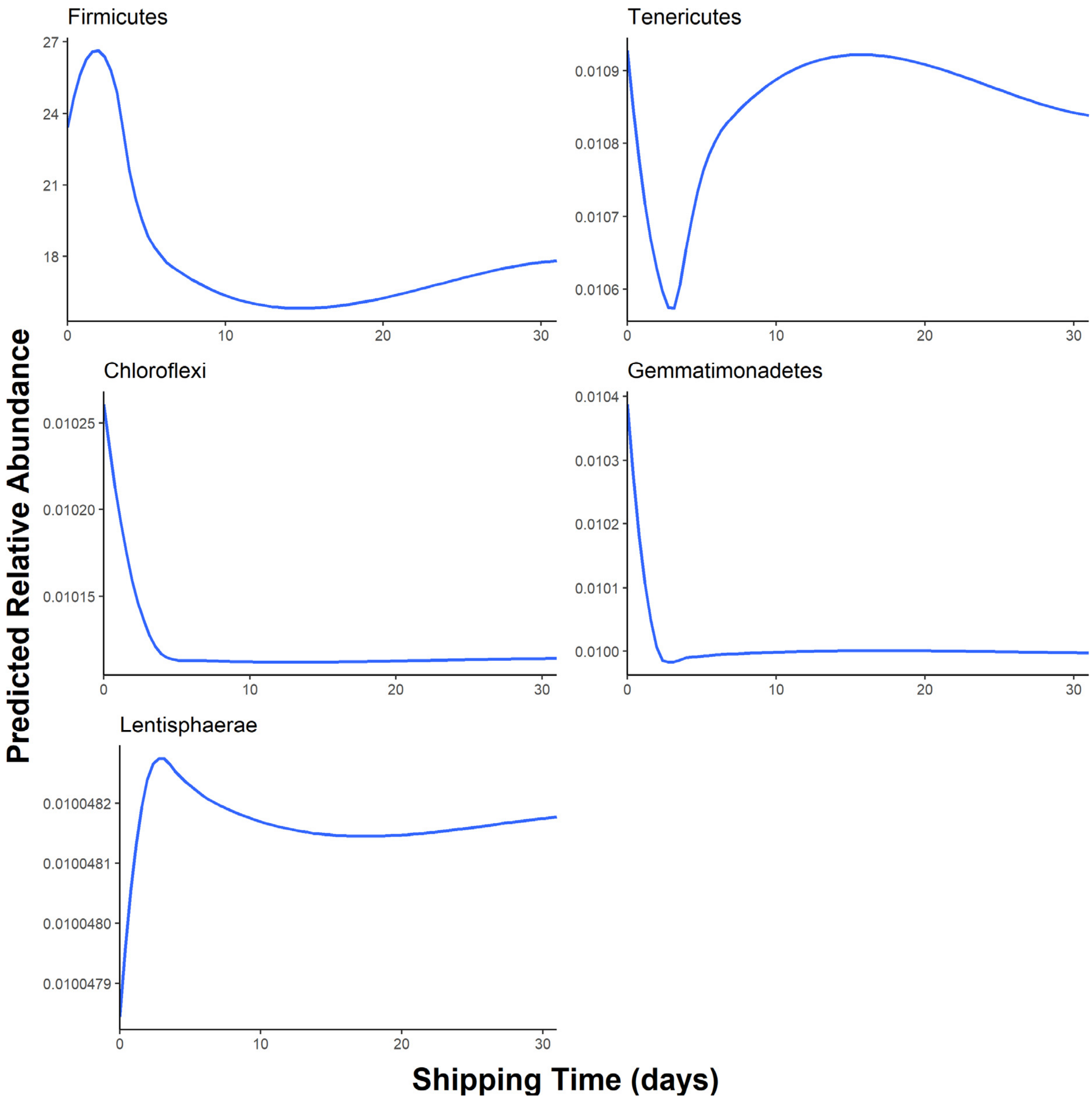

Figure S10. Phylum relative abundance predicted by sample shipping time. Only the phyla whose abundance was significantly predicted by age and sample shipping time are plotted.

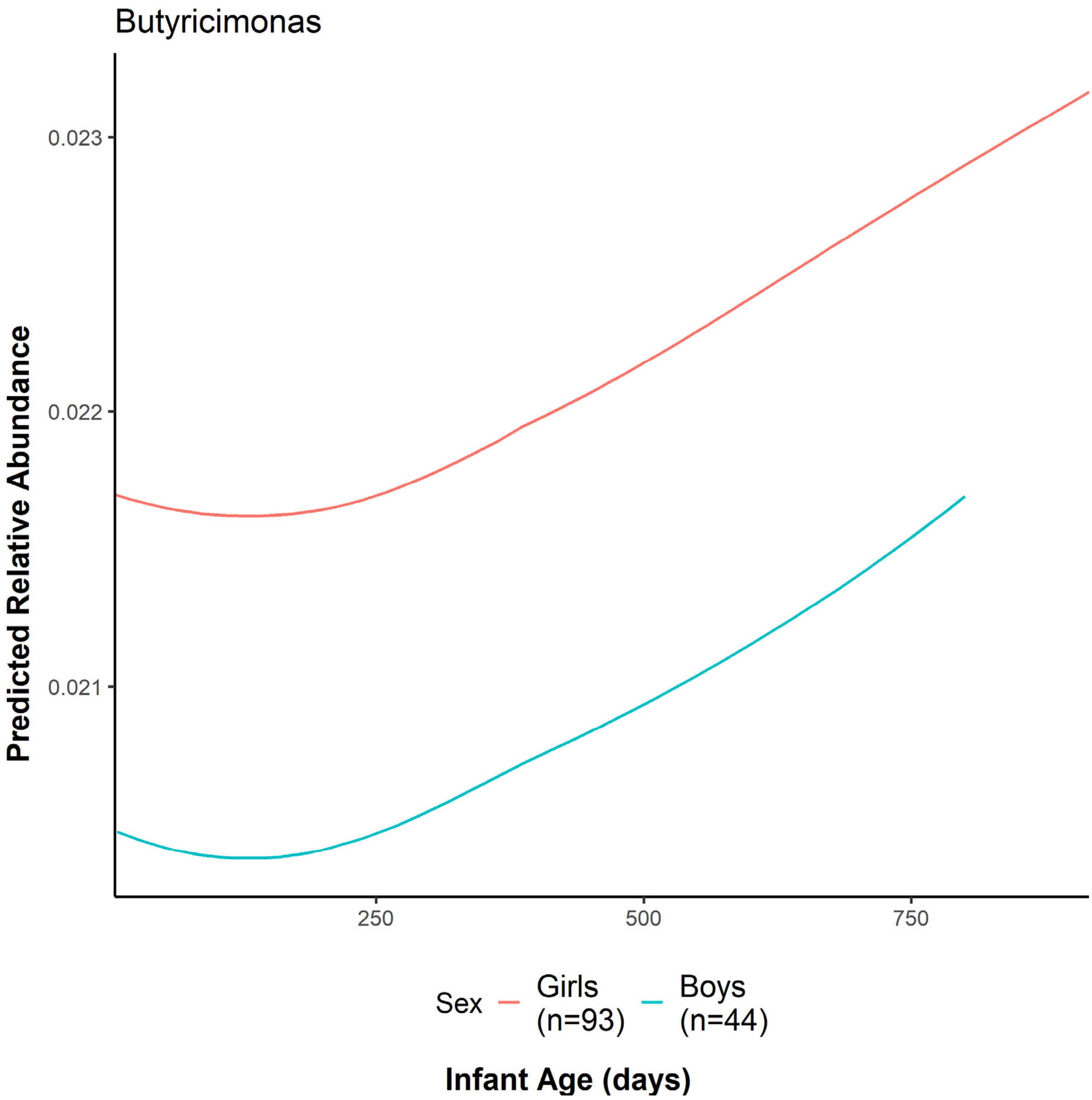

Figure S11. Genus relative abundance predicted by age and sex. Only the genera whose abundance was significantly predicted by age and sex are plotted.

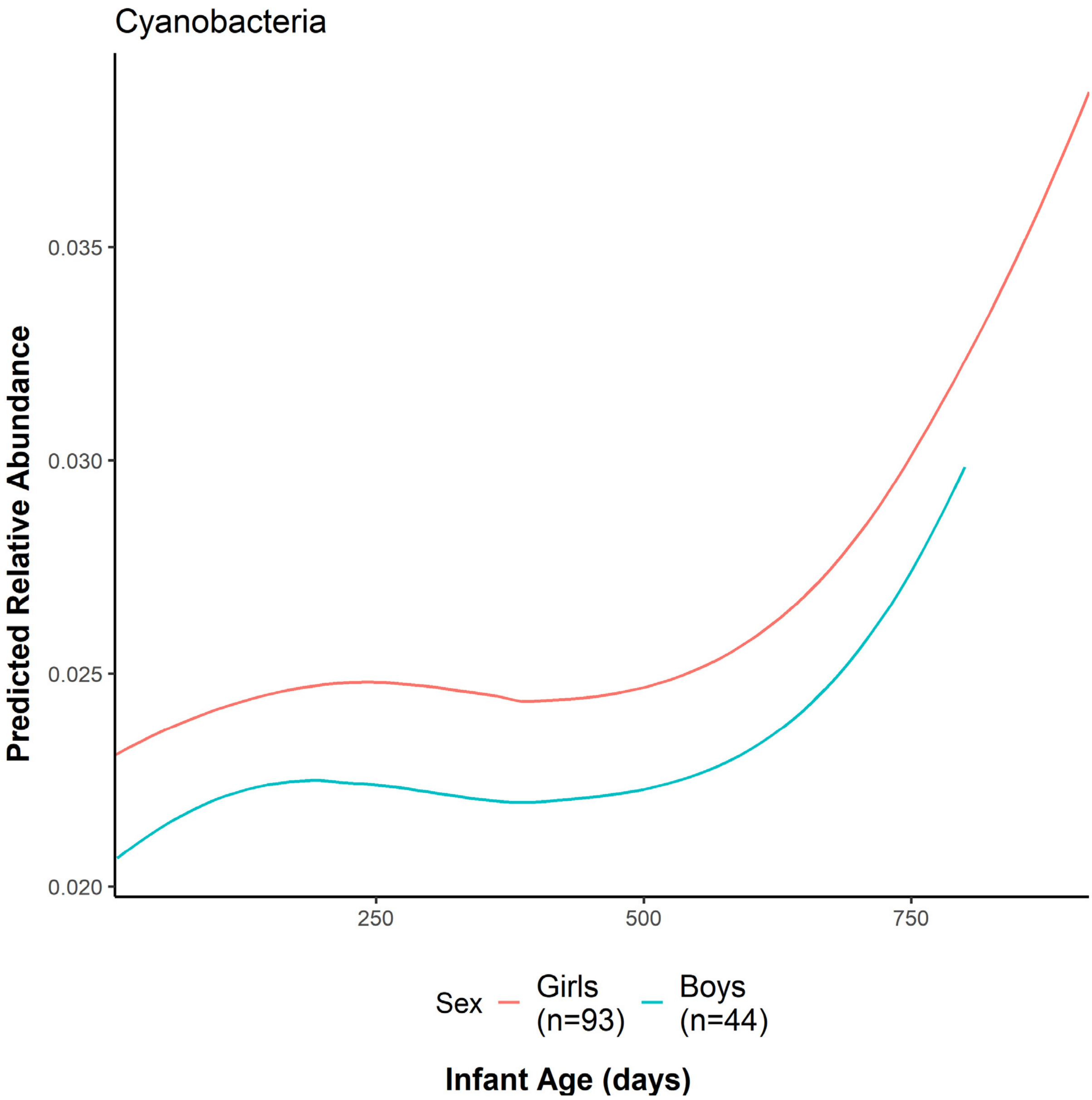

Figure S12. Phylum relative abundance predicted by age and sex. Only the phyla whose abundance was significantly predicted by age and sex are plotted.
